# Editorial: Spontaneous coronary artery dissection: pathology and pathophysiology

**DOI:** 10.3389/fcvm.2025.1710862

**Published:** 2025-10-29

**Authors:** Ivo S. Petrov

**Affiliations:** Department of Cardiology and Angiology, Acibadem City Clinic, Sofia, Bulgaria

**Keywords:** spontaneous coronary artery dissection (SCAD), FFR in coronary dissection, fibromuscular dysplasia (FMD) in coronary dissection, genetics in coronary dissection, indications of PCI for coronary dissection, echoCG and cardiac MR in spontaneous coronary dissection

**Editorial on the Research Topic**
Spontaneous coronary artery dissection: pathology and pathophysiology

## Introduction

Spontaneous coronary artery dissection (SCAD) is an increasingly recognized cause of acute coronary syndrome (ACS), particularly affecting young and otherwise healthy women. Unlike atherosclerotic coronary events, SCAD involves the formation of an intramural hematoma or intimal tear within the coronary artery wall, leading to lumen compression, myocardial ischemia, and, in some cases, sudden cardiac death. Although awareness of SCAD has grown, its etiology, genetic underpinnings, and optimal management remain incompletely understood, underscoring the need for continued investigation. This Research Topic, Spontaneous Coronary Artery Dissection: Pathology and Pathophysiology, brings together a collection of five diverse and high-quality articles that address the genetic, molecular, structural, and clinical dimensions of SCAD. The insights presented here contribute to a more nuanced understanding of SCAD and highlight opportunities for future translational research.

## Genetics and molecular pathways in SCAD

Two of the papers focus on the genetic and proteomic underpinnings of SCAD, reinforcing the hypothesis that SCAD may arise in genetically predisposed individuals through complex and multigenic pathways. In the first study, Casula et al., investigated the genetic architecture of SCAD in an Italian cohort using whole-exome sequencing (WES) and trio-WES approaches. Among 15 SCAD patients, they identified 37 rare variants in 34 genes, including likely pathogenic variants in COL3A1, COL1A2, and SMAD3—genes previously linked to connective tissue disorders (CTDs). Of particular interest, a premutation allele in the FMR1 gene was identified in two related patients, suggesting a potential novel mechanism involving mRNA toxicity and small RNA dysregulation. The study not only confirms the role of extracellular matrix dysregulation but also expands the potential genetic contributors to SCAD, including DROSHA, a gene implicated in miRNA processing (Casula et al.).

The second molecular study applied genome-wide association analysis and Mendelian randomization to identify circulating proteins associated with both SCAD and aortic aneurysm/dissection (AAD). The findings implicate extracellular matrix protein 1 (ECM1) and additional proteins involved in Ras signaling and lipid metabolism, supporting a shared vascular vulnerability between SCAD and AAD. The identification of ECM1 as a candidate biomarker and potential effector molecule in SCAD pathogenesis represent a promising avenue for translational research (Chai et al.).

## Fibromuscular dysplasia: a critical association

The association between SCAD and fibromuscular dysplasia (FMD) has been widely reported but remains incompletely characterized. In a mini-review, Eltabbakh et al., provides an update on the epidemiological, pathophysiological, and genetic overlaps between these two non-atherosclerotic vascular conditions. FMD prevalence in SCAD cohorts varies widely (25%–86%), likely due to differences in imaging modalities and screening practices. This review reinforces the importance of systematic screening for FMD in SCAD patients, not only to identify shared risk factors but also to explore whether FMD might serve as a predictor of SCAD recurrence. The review also points to underexplored areas such as the role of physical activity and long-term management in patients with dual pathology (Eltabbakh et al.).

## Monitoring and outcomes: clinical tools and challenges

Management of SCAD remains a clinical dilemma, with ongoing debate regarding the appropriateness of conservative therapy vs. percutaneous coronary intervention (PCI). A review by Krljanac et al., explores how echocardiography and cardiac magnetic resonance (CMR) can be employed to elucidate treatment determinants such as myocardial injury and left ventricular function in SCAD survivors. Given the heterogeneity in myocardial involvement and recovery, personalized imaging protocols may help optimize follow-up and risk stratification. This work underscores the need for more standardized post-SCAD monitoring and highlights the potential role of imaging biomarkers in predicting recurrence and guiding therapy (Krljanac et al.).

Finally, while not SCAD-specific, a retrospective study by Zhang et al., examining outcomes following fractional flow reserve (FFR)-guided therapy provides insights into broader vessel-related event prediction in coronary disease. The findings suggest that FFR-based strategies may reduce target vessel failure, although non-target vessel events remain a confounding factor. These data support the principle of physiologic guidance in coronary interventions, which could be even more relevant in SCAD patients with coexisting atherosclerotic disease (Zhang et al.).

## Conclusion and future perspectives

This Research Topic highlights the multifactorial nature of SCAD, with genetic, structural, and inflammatory components converging on a final common pathway of coronary artery wall disruption. Addressing all the remaining questions will require collaborative, multicenter efforts, combining genomics, imaging, clinical data, and longitudinal follow-up. Importantly, a greater focus on sex-specific cardiovascular research is needed, given the disproportionate impact of SCAD on women. We thank the contributing authors for their valuable work and the reviewers for their critical insights. It is our hope that this collection will inspire further exploration into the mechanisms and management of SCAD and contributes to better outcomes for patients worldwide.

